# Real-Time Remote Patient Monitoring and Alarming System for Noncommunicable Lifestyle Diseases

**DOI:** 10.1155/2023/9965226

**Published:** 2023-11-20

**Authors:** Htet Yamin Ko Ko, Nitin Kumar Tripathi, Chitrini Mozumder, Sombat Muengtaweepongsa, Indrajit Pal

**Affiliations:** ^1^Department of Information and Communication Technologies, School of Engineering and Technology, Asian Institute of Technology, Pathum Thani 12120, Thailand; ^2^Center of Excellence in Stroke, Faculty of Medicine, Thammasat University, Pathum Thani 10121, Thailand; ^3^School of Environment, Resources and Development, Asian Institute of Technology, Pathum Thani 12120, Thailand

## Abstract

Telemedicine and remote patient monitoring (RPM) systems have been gaining interest and received adaptation in healthcare sectors since the COVID-19 pandemic due to their efficiency and capability to deliver timely healthcare services while containing COVID-19 transmission. These systems were developed using the latest technology in wireless sensors, medical devices, cloud computing, mobile computing, telecommunications, and machine learning technologies. In this article, a real-time remote patient monitoring system is proposed with an accessible, compact, accurate, and low-cost design. The implemented system is designed to an end-to-end communication interface between medical practitioners and patients. The objective of this study is to provide remote healthcare services to patients who need ongoing care or those who have been discharged from the hospital without affecting their daily routines. The developed monitoring system was then evaluated on 1177 records from MIMIC-III clinical dataset (aged between 19 and 99 years). The performance analysis of the proposed system achieved 88.7% accuracy in generating alerts with logistic regression classification algorithm. This result reflects positively on the quality and robustness of the proposed study. Since the processing time of the proposed system is less than 2 minutes, it can be stated that the system has a high computational speed and is convenient to use in real-time monitoring. Furthermore, the proposed system will fulfil to cover the lower doctor-to-patient ratio by monitoring patients from remote locations and aged people who reside in their residences.

## 1. Introduction

Telemedicine along with the remote patient monitoring (RPM) technology can improve the healthcare coverage by allowing early intervention and identification of early disease symptoms [1]. Global health emergency in 2019 exposed the challenges and weaknesses in healthcare sector such as mandatory in-person visits, insufficient ratio of doctor-to-patients, and the risk of respiratory disease transmission [2]. Therefore, the normal healthcare operating system is shifted to remote monitoring of noninfected and noncritical patients to provide the available facilities to the infected and critical patients in 2019-2020 [2]. With the usage of RPM systems, patients are allowed to stay in their choice of location with real-time medical service [2]. Gradually, RPM systems are advancing and applicable for the detection of specified illness, early detection of disease symptoms, and generation of alert for the critical situations [2]. As the problem of increasing aging population is rising at the developed countries, the demand for the telemonitoring system will increase to cover the doctor shortage and lack of medical coverage in their rural areas also. Other advantages of telemonitoring systems are the availability to deliver the healthcare services to the rural locations with reasonable price and availability of preventive healthcare way by the continuous monitoring. Regular usage of RPM systems in wider service areas can reflect the health profile of the whole country [3], and the data received from the monitoring systems can further be processed for the health-related knowledge and research [4].

The advancement of telemonitoring systems is facilitated by the rapid development of affordable wireless communication devices, electronic medical record (EMR) systems, and the development in healthcare services of the recent decades [5]. As governments including that of Thailand and most other countries focus to develop a framework for driving digital technology-based healthcare provision, it is important to adapt the creative advantages of digital infrastructure in the strategy of RPM systems [6]. According to the Ministry of Public Health (MOPH) of Thailand [6], the eHealth strategy which started from 2015 with the cooperation of many stakeholders and different sectors such as public health is aimed at developing a smart healthcare system for the benefit of population. World Health Organization (WHO) defines eHealth as a cost-sustainable and safe way of information and communication technology (ICT) to distribute well-being-related services, knowledge, and research in developed and developing countries [4]. In Fifty-eighth World Health Assembly [4], WHO urges its member countries to develop a long-term strategy for developing and implementing RPM systems in different sectors of healthcare. Therefore, it is necessary and important to develop an affordable, equitable, and universal accessible eHealth infrastructure for the benefit of communities [4].

Existing remote patient monitoring systems have incorporated threshold values or rule-based algorithms for the vital signs and heart rate parameters [3, 5]. These existing systems are becoming promising due to the compactness of the system along with delivery of remote medical care at doorstep. Much research has been done to deliver the healthcare service by monitoring physiological parameters, laboratory exams, wearable sensors, or remote sensors [7]. As a success story of existing RPM systems, Portable Healthcare Clinic (PHC) is referred as “Doctor in Box” which aims to provide the primary healthcare services at the doorstep of the unreached populations by using the telemedicine technology [3]. The concept of the PHC is to provide regular healthcare check-ups for the early disease mitigation and to promote the preventive healthcare. In traditional healthcare settings, patients visit hospitals only after experiencing symptoms or illness, often resulting in delayed diagnosis and increased disease severity [3]. The PHC system is equipped with a complete pack of medical sensors, an Internet-enabled tablet/pc, trained medical workers, an online data server, and the remote call canter for the doctor [3]. Medical workers perform the patient check-ups by using the medical sensors and fill in the PHC application manually [3]. Then, doctor can see the patient's information and make a video call with patient if further investigation is required. Following this, doctor prescribes by writing notes under patient's medical record in PHC application [3]. Each input parameter is then classified into four categories: green for healthy, yellow for caution, orange for affected, and red for urgent [3]. The PHC system is only aimed for the delivery of medial data and providing end-to-end communication framework between medical doctors and patients. The system does not incorporate with any decision support tool or machine learning-assisted alarming system.

As telemedicine is applied to provide care for the cardiac patients, intuitive healthcare monitoring system by using Zephyr BT wireless heart rate sensor is developed by using mobile application and web interface technology in [5]. Like PHC [5] and other remote care systems, user needs to register in mobile application by providing basic patient information [5]. Then, the sensor is worn by patient and linked by a mobile phone to send information about blood pressure, respiratory rate, and temperature to web server [5]. The data is fed into web server, and fuzzy logic is then applied to extract information about patient's condition (normal, bradycardia, tachycardia, two stages of hypertension, fever, hypothermia, and hypotension). Healthcare providers can access the web interface to see the analysis outcomes and alert messages based on the patient's status [5]. Their proposed system has alarm message features where healthcare providers will see alarm message if patient is in tachycardia and bradycardia condition [5]. Distributed computing system with IoT is used to develop healthcare monitoring function application for chikungunya virus to know the outbreak earlier [8]. The authors applied different types of sensors to collect various data; for example, body sensors are used to collect health data (fever, ache, rashes, headache, vomiting, headache, and muscle ache). Water quality detector is applied to collect the environmental data (quality of water, temperature of air, humidity, CO_2_, and mosquito density), RFID tag sensor collects prescription data (dosage, type, form, and strength), GPS sensor is used for locational information, and climate detector sensors are used for meteorological data (max temp, min temp, rainfall, and humidity) [8]. Each patient is classified as infected or uninfected using the fuzzy C-means (FCM) method. If the patient is categorized to be infected, the fog layer sends an alert message to patient's contact number, and at the same time, Outbreak Risk Index for every patient is calculated to check the possibility of transmission [8].

As RPM systems are targeted to provide support to control specific diseases, [9] proposed a telehealth platform to provide annual dilated eye check-ups for the diabetes patients located in the rural Arkansans, United States of America [9]. Teleretina program is based on the image data, and the captured images are transferred to the head station located in “University of Arkansas for Medical Sciences” (UAMS), Harvey and “Bernice Jones Eye Institute” (JEI), for diagnosis and consultation for medical treatments if necessary [9]. The authors stated that the developed system can aid in reducing the number of missing eye exam in diabetes patients per year [9]. Applications for telemonitoring can be used in a variety of ways, one of which is to retain personal health information [10]. Users can acquire self-management to take care of their health as well as maintain track of healthcare factors [10].

In the current research, the proposed system is modelled by using MIMIC-III clinical healthcare database data. An application (IOS and Android) is developed by using Flutter and Spring Boot, where XAMPP MySQL is used for the data storage server. The collected physiological parameters (body temperature, systolic blood pressure, pulse/heart rate, respiration rate, blood sugar level, body mass index, and oxygen saturation (SpO_2_)) are the independent variables, and the status of the patient (normal or abnormal) is the dependent variable of the presented study. The trained logistic classifier model is converted into tflite (TensorFlow Lite) file to embed into the flutter application. The tflite-flutter package is used to embed the TensorFlow classification mode [11]. As soon as the user enters the vital signs into the mobile application, the embedded machine learning model will compute the Early Warning Score (EWS) and classification of patient's status (normal/abnormal) based on the patient's physiological parameters.

The scientific contributions of this article are as follows:

- New alerting system is designed and tested by using EWS system, the machine learning models, and MIMIC-III dataset to monitor the patient's health status with manual input physiological parameters
- National Early Warning Score 2 (NEWS2) is computed to locate the patient's health situation and considered as one of the factors to generate the alert
- Resting heart rate is considered as one of the factors to generate the alert
- Different machine learning models are trained and tested to generate alert based on the patient's status

The current study directed the lack of the reliable analysis systems to avoid the alarm fatigue and the incorporation of non-medical-grade sensors for the monitoring process in the RPM systems. As the independent factors of the analysis model covered the parameters required to monitor the noncommunicable diseases, the proposed approach can be also utilized to screen the patients with underlying noncommunicable diseases. In this proposed study, we propose a real-time supervising and alarming system comprising with machine learning algorithm, mobile application, and web interface to fill the gap of current remote patient monitoring system. The proposed system can analyse the data with the embedded machine learning model to generate emergency alerts based on analysis outcome to inform the physician if there is a necessity of check-up or examination. The rest of the article are organized as follows: Section 2 presents the materials and methods.  Section 3 presents the proposed experimental setup. Experimental analyses are presented in Section 4, and Section 5 includes the conclusion of the research findings.

## 2. Materials and Methods

The study proposed an end-to-end remote monitoring framework to monitor patient's physiological parameters in real time with alarming system. The proposed system is aimed at providing medical care facilities to patients. The system is designed to reduce the paperwork and unnecessary follow-up hospital visits, and it is designed to use without complex setup of sensor devices. Age, body temperature, blood pressure, oxygen saturation, respiration rate, body mass index, pulse rate, heart rate, and blood sugar level data are used to detect the current health status of patients, and the long-term monitoring of previously stated physiological parameters can provide the early detection of life-threatening events (e.g., hypertension, hyperthermia, and arrhythmia) or disease symptoms. The developed system is designed with two user interface systems: mobile application for the patient side and the web interface for the doctor side [5]. But different from other existing systems, the system does not incorporate with sensor devices to provide nontechnical user-friendly system and to achieve the accurate measurement data from the medical-grade sensor devices. The detailed explanation of system design architecture, components, processing, EWS computation, and alarm generating system is presented in the following section.

### 2.1. System Architecture

**Figure 1:**
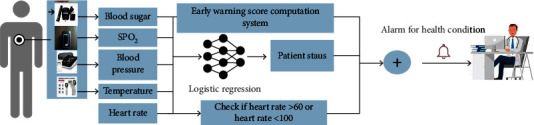
System diagram of proposed system.

The architecture of the proposed study is composed of two tiers such as a patient interface which is a smart phone and a doctor interface which is a website as shown in Figure 1. The first tier consists of an android smartphone with developed mobile application interface to fill out the user's basic information along with vital signs parameters to be monitored. The communication between an android application and the web interface will be performed over mobile Internet network, GPRS, or Wi-Fi networks. Additionally, the mobile application has the capability of body mass index computation, NEWS2 computation, and alert generation based on the user's health status. For the analysis of patient status based on the input parameters, logistic regression classifier is selected based on the performance comparison among several machine learning classifiers. In the website interface for medical practitioner, data imported into MySQL database will be extracted and the data from multiple patients will be reported along with results, patient's current location, and alerts. In this study, users are required to enter five vital signs parameters (pulse rate, body temperature, oxygen saturation, blood pressure, blood sugar level, and pulse rate) by using the sensor devices. The core of the proposed study is to foster a reliable and applicable decision support system to provide healthcare services for the patients with limited access to the medical visits by measuring the parameters required in real-time medical investigations.

Body temperature is the first parameter required in the vital signs monitoring of the proposed study, and it can vary based on weather, time of test, gender, and recent activity. If the patient is coming out from the very cold place, the taken temperature will be low, and if the patient is coming at afternoon, the taken temperature can be high [12]. The second parameter is the heart/pulse rate, and rate of heartbeat per minute is defined as pulse. It is also useful in indicating the workflow of heart and the strength of the blood flow. The values can be changed because of exercise, fever, injury, and feelings. The outcome of blood pressure measurement result includes systolic blood pressure and diastolic blood pressure. In the early warning score computation system, only systolic blood pressure measurement is used while both blood pressure measurements are used in the proposed system. In systolic blood pressure value, the result represents blood vessel pressure that was caused by the rate of blood flow control by the patient's heart in arteries to the other parts of the body [13]. Diastolic blood pressure value represents the pressure of the blood vessels when the heart pauses to squeeze and releases the blood flow, in other words, the moment when the heart is filled with blood and absorbs oxygen [13].

Oxygen saturation (SpO_2_) represents the oxygen saturation which is the fraction of oxygen-carrying haemoglobin and non-oxygen-carrying haemoglobin. It is important to monitor since the body needs to maintain SpO_2_ to prevent from malfunctioning [14]. Blood sugar level provides information about the patient's blood sugar level, and based on the results, medical practitioners can examine that the blood sugar level is low, high, or good. This value is mandatory for monitoring diabetes diseases [15]. The American Diabetes Association suggested people with underlying disease “diabetes” to check the blood glucose level three times per day at least. The normal range of blood sugar level is differed based on the range and the time of test (premeal or postmeal). The proposed system allows users to use any medical-grade sensor devices for the stated parameter measures but one thing to keep in mind is the measurement accurateness of the sensor devices has huge impact on the outcome of the parameters in the RPM systems. Hence, it is important to select the accurate and high-quality medical sensor devices for the early investigation and regular monitoring of underlying health condition [5].

### 2.2. National Early Warning Score (NEWS/EWS)

National Early Warning Score (NEWS/EWS) [16] is established by the “Royal College of Physicians” to improve the examination and reaction to the failure of medical deterioration of physiological parameters in inpatient. Different score systems, including the Modified Early Warning Score (MEWS), Pediatric Early Warning Score (PEWS), and National Early Warning Score (NEWS) 2, are selected to employ based on a particular type of patient visits (emergency, surgical, prehospital, and surgical) [1]. National Early Warning Score (NEWS) 2 is developed in 2012 and updated in 2017 to apply in acute and ambulance settings, and it has seen widespread uptake across the National Healthcare System (NHS) of the United Kingdom. Static EWS systems assign a score to each vital sign parameter, computation is performed for individual vital sign records, and presume the vital record as equal distribution [16], as shown in [2]. NEWS2 is a simple combined scoring system in which a score is assigned to physiological parameters as presented in Table 1, and the score of six measurement parameters is summed [17]. The summed score is used to determine the status of patient as presented in Table 2. Detection and estimation of patient clinical deterioration by computing NEWS with artificial intelligence [1] is the emerging area of machine learning for the specific type of clinical population.

### 2.3. Resting Heart Rate

Resting heart rate (HR) is a simple medical variable and is one of the risk assessment factors for patient after acute coronary syndromes [18]. It is included in computation of risk score such as “Global Registry of Acute Coronary Events risk prediction” score, “Myocardial Infarction Risk” score, and “Cooper Clinic risk index for overall mortality” [18]. Therefore, the proposed study includes HR as an analytical variable and latent medicinal target as stated by [18]. Recent studies found that HR is an independent indicator of cardiovascular and all-cause of mortality in mean and women with/without underlying cardiovascular diseases [18]. Therefore, including the HR factor in alarm generation model will provide assessment for cardiorespiratory fitness and cardiac function [18].

### 2.4. Machine Learning Algorithms

In this section, the theoretical explanation about the machine learning classifiers which are selected to benchmark in this study will be explained. Six machine learning classifiers have been used in this study to classify the medical deterioration with the developed system design.

Random forest (RF) is an ensemble learning technique-based machine learning classifier which is composed of a large collection of decision trees [16, 19]. The output of the RF is made by the voting process from each decision tree, and the output class that receives the major votes will select the outcome [16].

Logistic regression (LR) is a statistical method based on the probability applied for the classification and regression purposes [16]. The core of the LR is the sigmoid function which can be presented as below:

(1) $$\begin{array}{c} f\left(x\right)=\frac{1}{e^{-x}}, \end{array}$$

where *e* is the Euler number and *x* is the normalized value from 0 to 1 [16].

Decision tree (DT) machine learning classifier is a probabilistic model for regression and classification challenges [16]. In DT classifier, a random sample is input to the tree with a linear process of conditional statements. For individual nodes in the tree, a randomness level or entropy is applied to evaluate the capability of class agreement. The entropy function is stated as below:

(2) $$\begin{array}{c} E\left(s\right)=-\overset{J}{\underset{j=1}{\sum }}P_{j}\log_{2}P_{j}, \end{array}$$

where *P*_*j*_ is the probability of agreement with class at each node of the decision tree [16]. Later, DT model compares the level of entropy to derive the information gain which is given by the following equation:

(3) $$\begin{array}{c} G\left(S,S_{i}\right)=E\left(S\right)-\overset{I}{\underset{i=1}{\sum }}\frac{\left|S_{i}\right|}{\left|S\right|}\;H\left(S_{i}\right), \end{array}$$

where *E*(*S*) is the entropy of the dataset, *H*(*S*_*i*_) is the entropy of the subset of the input dataset, and |*S*_*i*_|/|*S*| is the length coefficient of a subset by the length of entire dataset [16].

XGBoost classifier is known as a scalable end-to-end tree boosting system which is suitable for sparse information and weighted quantile sketch for estimate tree learning [20]. XGBoost is called “Extreme Gradient Boosting” [20], and it is based on the supervised learning approach. XGBoost classifier can provide ensemble tree boosting for complex data science problems efficiently with less processing time [20].

K-nearest neighbor classifier is a supervised classification algorithm, and it is based on the voting method which attempts to classify what group a feature data is included [21]. The voting technique is performed by finding the distance between the new data to be classified and all data in the training dataset, selecting the specified number examples (*K*) which is the closet to the new input data [21]. It is popular because of its simplicity and simple to implement. It has one major drawback which is its performance depends on the size of the dataset.

Support vector machine (SVM) is a supervised machine learning algorithm which can use for classification and regression problems [22]. SVM builds a hyperplane or set of hyperplanes in a high or infinite dimensional space, which can be employed for classification, regression, or other tasks [22]. Spontaneously, a good classification is achieved by the hyperplane that has the largest distance to the nearest training data points of any class (referred as functional margin); meanwhile, in particular, the large margin can lessen the generalization error of the classifier [22]. The selected parameters of each classifier are presented in Table 3.

### 2.5. Model Evaluation Procedure

**Figure 2:**
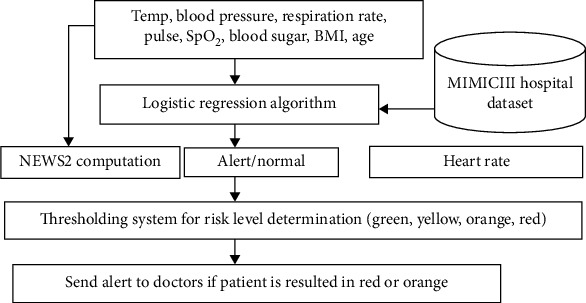
Alerting system based on the heart, blood sugar, and vital signs parameters for noncommunicable diseases.

The machine learning classifiers are trained, validated, and tested with MIMIC-III dataset, and it is required to evaluate the model's performance by computing the evaluating metrics [24]. The trained machine learning classifiers are applied to perform binary classification (normal/abnormal) from the patient's physiological parameters, as shown in Figure 2. The confusion matrix is firstly computed to compute the accuracy, recall, precision, F1-score, and other mean square error computation [24].

(4) $$\begin{array}{c} \text{Confusion matrix}=\left[\begin{array}{cc} \text{true positive}\left(\text{TP}\right) & \text{false negative}\left(\text{FN}\right)\\ \text{false positive}\left(\text{FP}\right) & \text{true negative}\left(\text{TN}\right) \end{array}\right], \end{array}$$

where TP stands for the count of correctly identified positive samples, FP stands for the count of correctly classified negative samples, FN stands for the samples incorrectly classified as positive, and TN stands for the samples incorrectly classified as negative [24].

Accuracy is computed by ratio of the accurately classified samples and the total sample counts in the dataset [24]. The recall which is known as the sensitivity or true-positive rate (TPR) is computed as the fraction between correctly classified positive samples and all samples assigned to the positive class [24]. Precision is defined as the fraction of the retrieved samples which are relevant and is evaluated as the proportion between correctly classified samples and all samples assigned to that class [24]. F1-score is the harmonic mean of precision and recall, meaning that it corrects extreme values of both [24]. Mean absolute error (MAE) and root mean square error (RMSE) of the machine learning models are also computed. MAE measures the average magnitude of the errors in a set of forecasts, without considering their direction [25]. Root mean square error or root mean square deviation is one of the most used measures for evaluating the average magnitude of the error [25]. K-fold cross-validation process with *K* = 10 is also performed to validate the robustness of each classifier.

## 3. Experimental Setup

This section will discuss about the dataset, preprocessing, and classification methods applied in this proposed study. In Figure 2, the experimental design of the proposed study is presented.

### 3.1. Dataset

The MIMIC-III database (version 1.4, 2016) is a freely accessible intensive clinical care dataset covering deidentified statistics on 46,520 patients and 58,976 admissions to the intensive care unit (ICU) of the Beth Israel Deaconess Medical Centre, Boston, USA, from 1 June 2001 to 31 October 2012 [26, 27]. The dataset provides complete data, such as demographics, admitting notes, “International Classification of Diseases-9^th^” revision (ICD-9) diagnoses, laboratory exams, prescriptions, care trials, discharge reports, fluid balance, vital parameter measurements measured at the bedside, records of care providers, radiology exam results, and survival data [27]. For the data selection process, the proposed study used the dataset developed by [27] with the selection of 1,177 adult patients out of 13,389 patients.

### 3.2. Preprocessing

Variables with missing data are handled with data imputation where the absent data are imputed by the mean value for the patient group [27]. Data normalization procedure is excluded in [27] and the proposed system as the developed model will be embedded in mobile application to do real-time analysis of user filled vital signs values.

### 3.3. Machine Learning-Based Alerting System

Early recognition of clinical deterioration and alert generation functions are the critical part of remote patient monitoring system to reduce morbidity and mortality of patients [16]. The current challenging problems in the development of remote clinical deterioration alerting system are the increasing usage of remote monitoring systems with the increased volume of physiological data [16], alarm fatigue caused by the low accurate threshold based alarming system, and the lack of combination with high performance machine learning model and application development. With the development of Flutter framework and machine learning applications, an intelligent machine learning-based alert generation system is embedded into the mobile application. In this proposed study, 1177 data points from MIMIC-III clinical dataset are used with six different scalable machine learning methods. 70% of dataset (824 records) is used as the training and validation datasets, and the rest of 30% (352 records) is used for the testing purpose.

**Figure 3:**
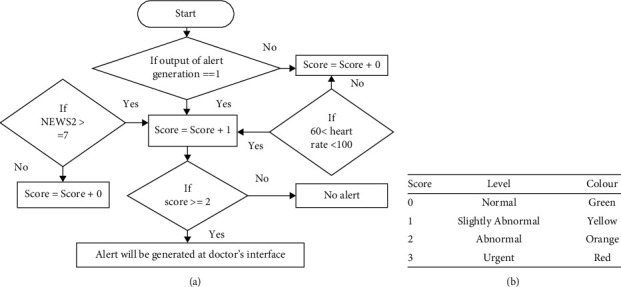
(a) Thresholding system for the risk level determination, and (b) the level and colour indicating the alert of eHealth remote patient monitoring system.

The alert will be computed by using the aggregated scoring system. The alert score will add one if the patient risk identification system outcome is abnormal. Otherwise, the alert score will add zero. NEWS2 will be computed by using the systolic blood pressure, respiration rate, body temperature, pulse rate, and oxygen saturation (SpO_2_). For the proposed system, the consciousness value will set as “alert” and air/oxygen parameter will set as “air” because the proposed system is designed to provide patients which are under noncritical conditions. The patients who are in pain, unconscious, or have problems with breathing should go and meet with medical practitioners immediately. If the NEWS2 score is greater than 7, the alert score will add one again. Otherwise, the alert score will add zero. The alert score will add one if the heart rate is within the normal range (60 ≤ heart rate ≤ 100 beat per minutes) [18]. The patient with zero score will be considered as green, the patient with one score will be considered as yellow, the patient with two scores will be considered as orange, and the patients with three scores will be considered as the requirement of urgent care. Therefore, the situation of patient's level will be determined by using green, yellow, orange, or red colour. Alerts will be generated for patients with red or orange levels, and it will show up at the web interface for medical practitioners. The threshold alarming system of the proposed study is presented in Figure 3. The aggregated outcomes of the patient risk identification system, NEWS2 score, and heart rate thresholded system will be used to indicate the patient's status into green, yellow, orange, and red as shown in Figure 3(b).

The proposed system will generate alert based on the analysis of blood sugar level along with body temperature, blood pressure (sys/dia), respiration rate, age, gender, and body mass index data. The logistic regression classification algorithm will be used to classify a patient's underlying status (normal or abnormal) by using the collected data. Logistic regression classifier is selected based on its highest accuracy, F1-score, and recall scores with lowest mean square error. NEWS2 computation will be performed by using patient's physiological parameters, and patients with NEWS2 score > 7 will be considered as abnormal. The heart rate less than 60 or greater than 100 will be considered as abnormal condition. The alert will be generated based on the outcome of patient condition analysis system, NEWS2 score, and the heart rate as shown in Figure 3(b).

### 3.4. Development of Mobile Application for the User Interface

**Figure 4:**
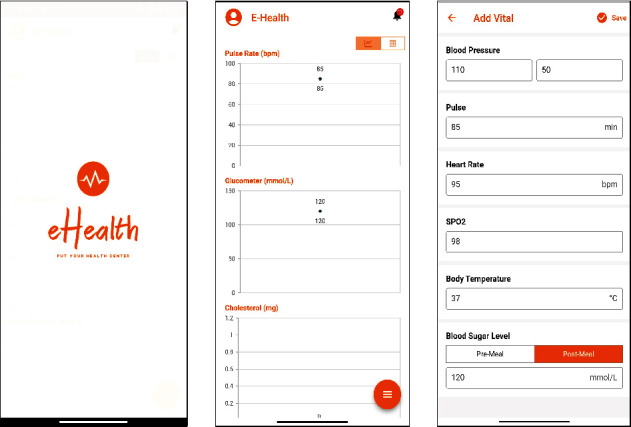
Application interface of patient user interface: vital signs chart and the vital signs filling form.

The development of mobile application is performed by using Flutter framework, Spring Boot, and Android Studio interface. The developed mobile application is required to install and register by the patients to act as an interface for the communication with medical doctors and getting medical services. For the first-time users, users are required to provide username, full name, password, mobile number, and gender information as the registration process. Before filling out the vital signs parameters, users are required to fill the basic information which are required in medical investigation such as weight, height, underlying diseases, blood type, and allergy information. Once the basic information is saved, the application will compute the body mass index information automatically. After that, users are allowed to fill in the vital signs parameters (as shown in Figure 4) such as body temperature, blood pressure, pulse rate, SpO_2_, blood sugar level, meal status of blood sugar level test, and last menstruation date for the female patients. After user clicked on the “Save” button, NEWS2 score along with the status of patient health condition will be computed with embedded machine learning models in the application. The filled vital information along with NEWS2 value and the alert score will be computed. Based on the alert score, the alert will be generated at the medical doctor interface.

### 3.5. Development of Web Application for the Care Provider Interface

**Figure 5:**
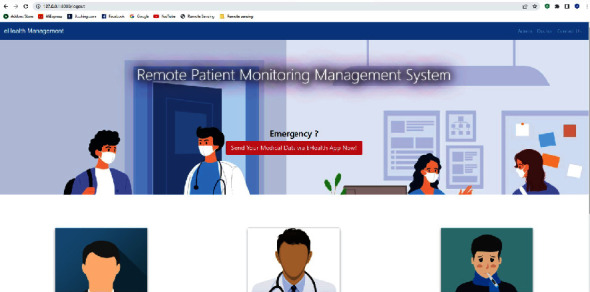
Home page of eHealth remote patient monitoring system.

The purpose of the web interface (Figure 5) is to allow admins and medical practitioners of the medical canter to view, get report about the patient's status, and diagnose the patient's physiological parameters instantly. The web interface is developed by using Python Django framework with Microsoft Visual Studio Interface. To provide data security and confidentiality, doctors are required to register first. After the registration process, doctors can log in with their username and password to use web interface as a medium of providing medical care to the patients.

#### 3.5.1. Modules of the Web Interface

**Figure 6:**
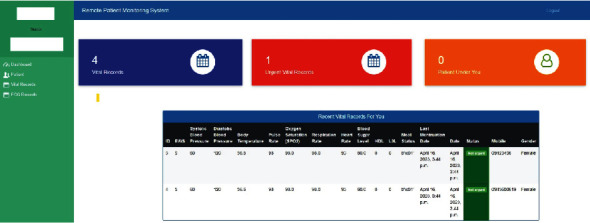
Doctor dashboard of eHealth remote patient monitoring system.

*(1) Doctor Dashboard*. This screen (Figure 6) will assist medical doctors to see the newly added vital signs records, vital signs records with abnormal status, and the total number of patient counts. After that, doctors can also see the updated vital sign tables of patients under his/her care. Urgent vital records will act as the alarming module for the patient's abnormal status.

**Figure 7:**
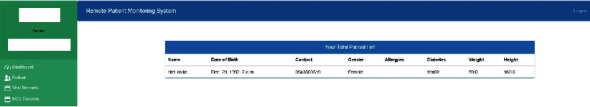
Patient module of eHealth remote patient monitoring system.

*(2) Patients*. This module (Figure 7) will provide complete information about the patient's basic and medical history information. Patients are required to register by using the eHealth mobile application.

**Figure 8:**
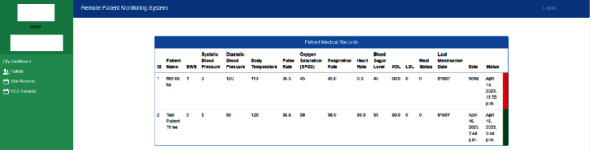
Vital record screen of eHealth remote patient monitoring system.

*(3) Vital Records*. In this module (Figure 8), all the vital information uploaded by the patients can be seen along with status of urgency. “Red” colour indicates for the patients with urgent status while “green” colour indicates for the patients with normal/stable condition.

**Figure 9:**
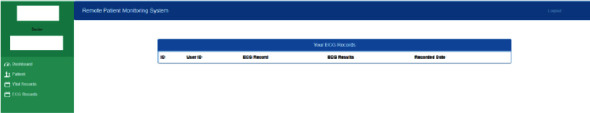
ECG record screen of eHealth remote patient monitoring system.

*(4) ECG Records*. In this module (Figure 9), all the ECG information uploaded by the patients can be seen along with the analysis results. This module is under implementation, and the screen requires to add the chart to present the uploaded ECG signal. The development of this module is out of scope for this proposed study.

### 3.6. Real-Time Implementation of the Proposed System

**Figure 10:**
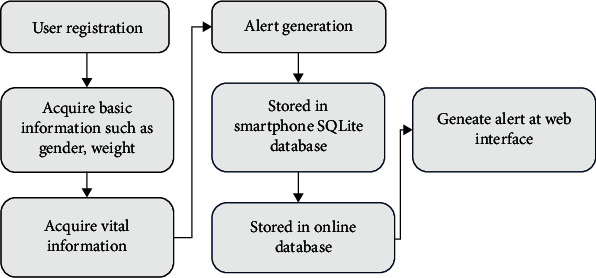
Flowchart of real-time remote monitoring system.

The real-time implementation of the proposed system is implemented by registering the user by using the developed Android application. The application will collect all the necessary information which are required in the hospital screen process. After that, user can input vital signs parameters by using the “Add Vital Signs” screen. The purpose of the proposed system is to send alarm message immediately after detection of abnormalities in the physiological parameters. The system will notify the level of patient condition into four levels: normal, slightly abnormal, abnormal, and urgent. The architecture of the proposed system can be seen in Figure 10.

#### 3.6.1. Alarming Response Time

The response time of the alarming mechanism is computed by the process of entering vital signs parameters, performing analysis on the mobile device, and generating an alert at the web interface by using the MIMIC-III clinical database. The response time is computed by using the two different networks on the same mobile device (Xiaomi 8 Lite). The tested networks are DTAC 4G mobile network and private/public Wi-Fi. The complete time to generate alert at the web interface took 18 seconds for all type of alerts, and the total of information filling and alarm generation took nearly 1 minute. The average alarm generation time is computed by the average value of 10 alarms in each alarm type and presented in Table 4.

## 4. Results and Discussion

The authors proposed a real-time remote monitoring system which is focusing on the analysis of the vital signs parameters, blood sugar level, and the heart rate. New alerting system design is proposed by computing the alerting score to classify patient's status into four categories. As the heart rate and the other cardiac parameters such as temperature and blood pressure [5] are examined in this proposed study, the implemented system can be used to monitor the heart prone patients also. Android application is implemented with simple user interface design to fill in and save clinical records of the patient which will be delivered to the web user interface using wireless communication. Web user interface is designed for the medical centre and the medical practitioners to alert the patient's status together with analysis results. The medical record data is stored in database server which will store and report the information back to medical centre's web interface, and eventually, physiological parameters of patients situated in inaccessible area are ended visible to the doctor locating in hospital. Furthermore, the realistic implementation of the proposed real-time monitoring system is investigated to verify the capability, accurateness, and performance of the system.

The proposed system is modelled by using MIMIC-III clinical healthcare database, and the statistical information of the patients is presented in Table 5. The research hypothesis of the study is “Finding the correlation of physiological parameters with the context of machine learning can enable the early, automatic and real-time alarming system.” The collected physiological parameters (body temperature, systolic blood pressure, respiration rate, blood sugar level, pulse/heart rate, body mass index (BMI), and oxygen saturation (SpO_2_)) are the independent variables, and the status of patient (normal or abnormal) is the dependent variable of the presented study.

**Figure 11:**
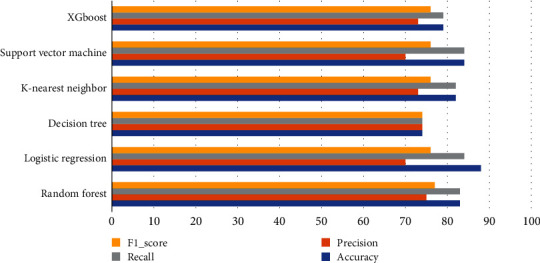
Comparison chart of trained classifiers.

The training process is performed by using random forest, logistic regression, decision tree, K-nearest neighbor classifier, support vector machine, and XGBoost classifiers. The performance of the classifiers is compared by using the confusion matrix indices, mean absolute error (MAE), and root mean square error (RMSE) as shown in Table 6. As compared in Figure 11, logistic regression classifier is outperformed among other classifiers with the highest accuracy and recall scores along with the lowest MAE and RMSE scores, and it is chosen for the proposed model as presented in Figure 11. Logistic regression model has the lowest precision score and highest F1-score when it is compared with other classifiers, but the system is focused to reduce the false alarm by finding the positive cases (high F1-score). In the proposed study, false-positive cases are more outweighed than the false-negative cases to reduce the rate of patients having undergone further screenings or using medication unnecessarily.

The dataset is trained and tested with K-fold cross-validation. In K-fold cross-validation process, the dataset is shuffled and divided into *K*th portions, one portion will be used as test data, and the rest will be used as the training data. In this study, we selected *K* = 10 for the cross-validation process and 7 is presented as the average accuracy of validation results. According to the results, the accuracy of most model increased when it is compared to Table 6 while the accuracy of logistic regression classifier decreased from 88% to 86.41%. Training with the variety of folded dataset provided the validation and robustness of the proposed system.

## 5. Conclusions

Noncommunicable diseases like diabetes, heart disease, and hypertension are becoming increasingly common [3]. By paying attention to early warning signs, it is possible to prevent these diseases and their succeeding complications. For the countries in the Southeast Asian region, disease prevention should be the way of combating the noncommunicable lifestyle diseases under circumstances of inadequate competence of expenditure on medical bills, the insufficient doctor-to-patient ratio, and the lack of establishment from authorities in some countries. For instance, public hospitals in Myanmar are operated by the government and relatively affordable compared to the private hospitals, although they are not completely free. However, using public healthcare services is highly inconvenient due to the limited number of bed availability and the low care provider-to-patient ratio. Therefore, prevention of diseases from spreading or at least detecting it at premature stage can benefit both government and community to save costs and human resources [3].

Another issue that is required to address is the low doctor-to-patient ratios in the developing countries, and it is impossible to increase the doctor-to-patient ratio immediately. Therefore, it is important to facilitate the medical practitioners to utilize the time of diagnosing patients [6] while reducing the number of patients by the preventive healthcare way [3]. Increasing interests in the usage of RPM system for the chronic and nonchronic patient care and the lack of reliability for the RPM system from the medical practitioner's side become challenges in the development of real-time cost-effective and high-efficiency RPM systems. As compared to the PHC [3] and other related works, the proposed system can provide the promising future for developing the eHealth infrastructure for Thailand and other Southeast Asian countries. The proposed system can provide the primary healthcare services for the unreached communities to address the common healthcare-related problems for the local people in Myanmar and Thailand.

The scope of the proposed system is to develop a real-time monitoring module to detect the early clinical deterioration detection for the abnormalities in the heart and blood sugar level along with physiological parameters using the wireless technology. The expected outcome of the developed system is to provide better healthcare service, to reduce the inpatient morbidity and mortality rates, and to reduce the effects of lifestyle diseases (heart and diabetes). In order to provide the robust alarming system to reduce alarm fatigue, new alarming system architecture is proposed and tested with several machine learning classifiers. According to the performance comparison presented at Tables 6 and 7, logistic regression model has the highest accuracy, lowest precision score, and highest F1-score when it is compared with other classifiers, but the system is focused to reduce the false alarm by finding the positive cases (high F1-score). Therefore, logistic regression model is selected as the classifier to embed into the mobile application for the alarm generation. The system targeted at elder patients or patients located in remote areas where the access to the medical service is limited, patients who are infected with transmittable diseases such as COVID-19, and the patients who are under monitoring stage for the rehabilitation.

Furthermore, the system is designed to provide the medical service under the circumstances of natural disasters such as earthquake or tsunami. The presented monitoring system is designed to compassionate with manual data entry by using medical sensors and the developed ECG sensor which will be used to collect the vital signs parameters, blood sugar level, heart rate, respiratory rate, and electrocardiogram signal. The extracted parameters will help to analyse the patient's medical status along with the analysis of abnormal heart condition. The development system is composed of mobile applications, sensors, and the web interface. The developed mobile application will be used for the patient's side to receive the ECG signal from the sensor, to fill in the basic information required for the medical screening, and to fill the vital signs parameters. The data from the patient's side will be analysed by the developed machine learning models and will transmit the data and results to the web server which will store the data for reporting purposes. The results and the original data will be presented at the doctor's web interface.

For further advancement of the current work, it is better to generate alert based on the consideration of previous medical record history. Currently, the alert is generated based on the one-time medical record which is practical for the outpatient visits but unpractical compared to the inpatient hospital care. To avoid the typing errors/user errors in the manual data entry [3], it is better to incorporate with Bluetooth-enabled medical-grade wearable sensors, and it can provide the continuous care for the patients under chronic conditions.

## Figures and Tables

**Table 1:** NEWS2 scoring system [17].

Physiological parameters	Score						
	3	2	1	0	1	2	3
Respiration rate (per minute)	≤8		9-11	12-20		21-24	≥25
SpO_2_ scale 1 (%)	≤91	92-93	94-95	≥96			
SpO_2_ scale 2 (%)	≤83	84-85	86-87	88-92			
Blood pressure systolic (mmHg)	≤90	91-100	101-110	111-219			≥220
Pulse rate (per minute)	≤40		41-50	51-90	91-110	111-130	≥131
Body temperature (degree Celsius)	≤35.0		35.1-36.0	36.1-380	38.1-39.0	≥39.1	

**Table 2:** NEWS2 thresholds and triggers [17].

Resulted aggregated NEWS2 score	Clinical risk
Score 0-4	Low
Score 3 in any individual parameter	Low medium
Score 5-6	Medium
Score 7 and more	High

**Table 3:** The choice of hyperparameters for each model [22, 23].

Machine learning models	Hyperparameters	Optimum values
Random forest [23]	The depth of the tree (*T*), number of tree models (*N*)	*T* = 3, *N* = 100

Logistic regression [22]	Confidence factor used for pruning (*C*), class weight adjustment (class weight), maximum iteration (max_iter)	*C* = 1.0, class weight = None, dual = False, max_iter = 100

Decision tree [22]	Confidence factor used for pruning (*C*), minimum number of instances of each leaf (*N*)	*C* = 0.25, *N* = 2

K-nearest neighbors [22]	Number of neighbors (*n*_neighbors), weight function used in prediction (weights)	*n*_neighbors = 5, weights = uniform

Support vector machine [22]	Confidence factor used for pruning (*C*), kernel type (kernel); maximum iteration(max_iter)	*C* = 1.0, kernel = ^“^linear^”^, max_iter = 100

XGBoost [22]	Depth of the tree (*T*), learning rate, number of estimators, gamma, and several tuning parameters	*T* = 3, learning rate = 0.1, number of estimators = 100

**Table 4:** Average alarm generating time.

Alarm generation	Alert generation time in Wi-Fi (seconds)	Alert generation time in DTAC 4G mobile network (seconds)	Total processing time (information filling + alarm generation) in Wi-Fi (seconds)	Total processing time (information filling + alarm generation) in DTAC 4G network (seconds)
Alerts (abnormal/urgent)	18	15	57	59

**Table 5:** Statistical data of MIMIC-III clinical dataset.

	Age	BMI	Heart rate	Blood pressure (systolic)	Resp rate	Temp	SpO_2_	Blood sugar level
Mean	74.05	30.188	84.57	117.99	20.8	36.68	96.27	148.79
Std	13.43	8.4305	15.929	17.24	3.98	0.602	2.285	51.098
Min	19	13.3468	36.000	75	11.14	33.25	75.91	66.67
25%	65	25.2769	72.54	105.5	17.96	36.29	95	114
50%	77	30.188	83.782	116.4	20.4	36.66	96.42	137.38
75%	85	32.1013	95.608	128.49	23.37	37.02	97.89	169
Max	99	104.97	135.7083	203	40.9	39.13	100	414.1

Std stands for standard deviation.

**Table 6:** Performance comparison of tested classifiers for patient risk identification system (Acc for accuracy, Pre for precision, and F1 for F1-score).

Classifier	Acc	Pre	Recall	F1	Mean absolute error (MAE)	Root mean square error (RMSE)
Random forest	83	75	83	77	0.172	0.42
Logistic regression	88	70	84	76	0.163	0.404
Decision tree	74	74	74	74	0.257	0.507
K-nearest neighbor	82	73	82	76	0.18	0.425
Support vector machine	84	70	84	76	0.163	0.404
XGBoost	79	73	79	76	0.209	0.457

**Table 7:** Accuracy assessment with K-fold cross-validation.

Classifier	Average accuracy (K-fold cross validation, *K* = 10)
Random forest	86.58
Logistic regression	86.41
Decision tree	76.72
K-nearest neighbor	84.54
Support vector machine	86.49
XGBoost	85.73

## Data Availability

Data supporting the findings of this study are available from the corresponding author on request.
